# A novel method for measuring acute thermal tolerance in fish embryos

**DOI:** 10.1093/conphys/coad061

**Published:** 2023-08-08

**Authors:** Zara-Louise Cowan, Anna H Andreassen, Jeremy De Bonville, Leon Green, Sandra A Binning, Lorena Silva-Garay, Fredrik Jutfelt, Josefin Sundin

**Affiliations:** Department of Biology, Faculty of Natural Sciences, Norwegian University of Science and Technology, Høgskoleringen 5, Trondheim, 7491, Norway; Department of Biology, Faculty of Natural Sciences, Norwegian University of Science and Technology, Høgskoleringen 5, Trondheim, 7491, Norway; Groupe de Recherche Interuniversitaire en Limnologie et en Environnement Aquatique (GRIL), Département de Sciences Biologiques, Université de Montréal, 1375 Av. Théres̀e-Lavoie-Roux, Montréal, H2V 0B3, Canada; Department of Biology and Environmental Sciences, Faculty of Natural Sciences, University of Gothenburg, Kristineberg Center, Fiskebäckskil, 451 78, Sweden; Groupe de Recherche Interuniversitaire en Limnologie et en Environnement Aquatique (GRIL), Département de Sciences Biologiques, Université de Montréal, 1375 Av. Théres̀e-Lavoie-Roux, Montréal, H2V 0B3, Canada; Department of Biology, Faculty of Natural Sciences, Norwegian University of Science and Technology, Høgskoleringen 5, Trondheim, 7491, Norway; Department of Biology, Faculty of Natural Sciences, Norwegian University of Science and Technology, Høgskoleringen 5, Trondheim, 7491, Norway; Department of Biology and Environmental Sciences, Faculty of Natural Sciences, University of Gothenburg, Kristineberg Center, Fiskebäckskil, 451 78, Sweden; Department of Aquatic Resources, Swedish University of Agricultural Sciences, Drottningholm, 178 93, Sweden

**Keywords:** climate change, critical thermal maximum, thermal physiology

## Abstract

Aquatic ectotherms are vulnerable to thermal stress, with embryos predicted to be more sensitive than juveniles and adults. When examining the vulnerability of species and life stages to warming, comparable methodology must be used to obtain robust conclusions. Critical thermal methodology is commonly used to characterize acute thermal tolerances in fishes, with critical thermal maximum (CT_max_) referring to the acute upper thermal tolerance limit. At this temperature, fish exhibit loss of controlled locomotion due to a temperature-induced collapse of vital physiological functions. While it is relatively easy to monitor behavioural responses and measure CT_max_ in larval and adult fish, this is more challenging in embryos, leading to a lack of data on this life stage, or that studies rely on potentially incomparable metrics. Here, we present a novel method for measuring CT_max_ in fish embryos, defined by the temperature at which embryos stop moving. Additionally, we compare this measurement with the temperature of the embryos’ last heartbeat, which has previously been proposed as a method for measuring embryonic CT_max_. We found that, like other life stages, late-stage embryos exhibited a period of increased activity, peaking approximately 2–3°C before CT_max_. Measurements of CT_max_ based on last movement are more conservative and easier to record in later developmental stages than measurements based on last heartbeat, and they also work well with large and small embryos. Importantly, CT_max_ measurements based on last movement in embryos are similar to measurements from larvae and adults based on loss of locomotory control. Using last heartbeat as CT_max_ in embryos likely overestimates acute thermal tolerance, as the heart is still beating when loss of response/equilibrium is reached in larvae/adults. The last movement technique described here allows for comparisons of acute thermal tolerance of embryos between species and across life stages, and as a response variable to treatments.

## Teaser

As climate change increases the frequency and severity of heatwaves, robust methodology to compare thermal sensitivity across life stages is needed. Here, we present a method for measuring acute upper thermal tolerance of fish embryos and show that resulting tolerance temperatures are comparable with those of larvae and adults.

Preprint: This manuscript has been deposited as a preprint to EcoEvoRxiv, DOI: 10.32942/X2V01R.

## Introduction

Climate change is increasing the frequency, duration and intensity of extreme weather events, such as heat waves, around the globe (e.g., Meehl and Tebaldi, 2004; Seneviratne *et al.,* 2014; Frölicher *et al.,* 2018; Frölicher and Laufkötter, 2018). Documented impacts of aquatic heat waves include species range shifts, widespread changes in species composition, and mass mortalities (e.g., Hughes *et al.,* 2018; Oliver *et al.,* 2018; Genin *et al.,* 2020; Smith *et al.,* 2023). Ectothermic organisms may be particularly vulnerable to extreme temperature fluctuations as their basic physiological functions are strongly influenced by environmental temperature (Deutsch *et al.,* 2008; Clark *et al.,* 2013). Mobile life stages might be able to move to more tolerable thermal habitats during extreme events. However, embryos that lack the ability to behaviourally thermoregulate can be restricted to thermally stressful locations. In addition, physiological differences might result in different thermal tolerance limits between life stages. For example, embryos often lack fully developed gills and circulatory systems, and are adapted to rely on oxygen diffusion through the egg, and subsequently skin, to sustain their metabolism (Rombough, 2005). Differences between life stages have been suggested to be caused by oxygen limitation, where embryos should have lower thermal tolerance due to lower oxygen transport capacity (Melzner *et al.,* 2009). Since no population is viable in the absence of functioning embryonic development, it is critical to evaluate responses of early life stages to thermal stress (Levy *et al.,* 2015; Telemeco *et al.,* 2017). Understanding the effect of increasing temperature across life stages is therefore vital to predict a population’s vulnerability to climate change.

A common and widely used method to quantify the acute upper thermal tolerance limits in aquatic animals, including fishes, is the critical thermal maximum (CT_max_) test (Becker and Genoway, 1979; Morgan *et al.,* 2018). The CT_max_ is generally measured as loss of equilibrium (LOE) in aquatic ectotherms following a steady increase in water temperature (Fry, 1947; Ern *et al.,* 2023). Measuring CT_max_ as loss of equilibrium is a non-lethal, robust method that is repeatable within individuals (Morgan *et al.,* 2018; Grinder *et al.,* 2020; O’Donnell *et al.,* 2020), and may not chronically impact the tested individuals (Morgan *et al.,* 2018). However, while it is relatively easy to monitor LOE and thus measure CT_max_ in juvenile and adult fish, this is not possible in embryos, which do not regulate their equilibrium when in the egg. Instead, median lethal temperatures (LT50), a protocol in which animals are exposed to constant temperatures and survival is measured after a given period (with LT50 referring to the temperature which is lethal to 50% of the individuals), is typically used (Fry, 1947; Dahlke *et al.,* 2020). The LT50 method presents several disadvantages, such as being more time consuming and requiring a greater number of test organisms for robust results. As the LT50 procedure uses death as an endpoint, it also may not be ideal or ethical in some contexts. Additionally, LT50 may not be comparable with CT_max_ because the duration of exposure and the absolute temperatures differ between the methods (Lutterschmidt and Hutchison, 1997; Ern *et al.,* 2023).

To circumvent the shortcomings of the LT50 method for estimating acute upper thermal tolerance, some studies have used the temperature at which the heart stops beating as an endpoint in fish embryos (Zebral *et al.,* 2018; Del Rio *et al.,* 2019). Methods to measure heart rate in embryos have used direct observation (e.g., Oulton *et al.,* 2013; Atherton and McCormick, 2015), and now also include automated software to analyse heart beat from videos of zebrafish (*Danio rerio*) embryos (Zebrafish Automatic Cardiovascular Assessment Framework, ZACAF; Naderi *et al.,* 2021). However, similar to LT50, the temperature at which the heart stops beating may not be directly comparable with CT_max_ based on LOE in other life stages, since the heart generally continues to beat after LOE is reached (Ekström *et al.,* 2016; Sandblom *et al.,* 2016). Given these difficulties, there is a lack of comparative data on thermal tolerance limits in the embryonic life stage in fishes. Using disparate techniques to measure CT_max_ makes comparisons across life stages difficult and potentially unreliable, which is an issue of great concern since such comparisons are critical to predict species vulnerability to climate change.

Spontaneous activity as well as responses to touch have been investigated in embryos of zebrafish (analysed from video recordings) (Kimmel *et al.,* 1974) and fathead minnows (*Pimephales promelas*) (spontaneous activity, analysed using automated image-tracking software) (Crowder and Ward, 2022). Since spontaneous embryonic movement can be quantified, we hypothesized that cessation of activity can represent a practical and comparable proxy for LOE in this life stage. Here, we developed a practical and robust method for estimating acute thermal tolerance in late-stage fish embryos based on this movement behaviour. We also validated the method by comparing it with other life stages. The method was tested on two temperate marine species: the three-spined stickleback (*Gasterosteus aculeatus*) and the black goby (*Gobius niger*). Both species are benthic spawners that provide paternal care, but the embryos differ dramatically in size, allowing us to test the applicability of the method between phylogenetically distant species and egg sizes. We demonstrate that this method is comparable with established protocols for measuring acute thermal tolerance in larval and adult life stages, thereby providing a possibility to compare CT_max_ across life stages.

## Materials and Methods

Experiments were conducted in May to July 2022 at the University of Gothenburg’s Kristineberg Center for Marine Research and Innovation (58° 14′ 59.1″, 11° 26′ 41.1″) by the Gullmar fjord (Sweden).

### Collection and maintenance of study species

Adult three-spined sticklebacks (*G. aculeatus*; hereafter “sticklebacks”) were collected using a beach seine pulled by hand in a bay of the Gullmar fjord (58° 14′ 33.8″ , 11° 28′ 07.5″) between 12–23 June 2022. Individuals were transported to the research station, where they were kept in groups of ~ 40 in glass holding aquaria (60 × 38 × 36 cm [L × W × H], water level 30 cm) with artificial plastic plants provided for shelter and sand as bottom substrate. Aquaria received flow-through, filtered seawater pumped into the station from a depth of 7 m (surface water supply). Temperature and salinity in the holding tank initially followed natural conditions in the area (means ± SDs: temperature, 14.8 ± 0.19°C; salinity, 26.3 ± 0.36 PSU). Starting on 16 June, the water temperature was increased (thermo-regulated) to a target temperature of ~ 18°C over a period of two days (actual mean ± SD temperature during holding: 18.08°C ± 0.11°C, data collected and averaged per day from two RBRsolo^3^ temperature loggers placed in separate tanks in the same flow-through system).

Adult black gobies (*G. niger*) were collected near the research station in the Gullmar fjord (58°14′57.4″N 11°26′49.6″E) in May to June 2022, using baited traps (mesh crab cages) with a soak time of 1 hour, and using a beach seine pulled by hand in bays of the Gullmar fjord within 2 km of the research station (58°14′55.5″N 11°26′50.8″E; 58°15′08.2″N 11°27′55.6″E). Individuals were immediately transported to the research station, where they were kept in either of two communal holding tanks, together with goldsinny wrasse (*Ctenolabrus rupestris*) (306 L, 80 × 75 × 51 cm) or with goldsinny and corkwing wrasse (*Symphodus melops*) (1350 L, 275 × 79 × 62 cm). Algae and cut PVC pipes lined with acetate sheets were provided for shelter for all fish. Since fish in the communal holding tanks spawned intermittently, the pipes were checked regularly for eggs. When a spawning had occurred, the PVC pipe with acetate sheet on which the eggs were laid, together with the male guarding them, were moved to separate aquaria and the embryos were also included in the presented study (but see information regarding dedicated spawning tanks described below). All holding tanks received flow-through, filtered seawater (surface water supply). For the first 29 days of holding, the rising spring temperature followed natural conditions (means ± SDs: temperature, 13.11 ± 1.46°C; daily average data from the continuous monitoring system at the research station, 17 May to 15 June 2022: http://www.weather.loven.gu.se/kristineberg/en/data.shtml), and was thereafter controlled at 18.08°C ± 0.11°C (mean ± S.D.) for the 20 individuals kept in spawning tanks (see below).

The photoperiod was set to 18 h light and 6 h darkness for all fish in all experimental rooms, to mimic natural conditions, regulated by lights on a timer from 05:00 to 23:00. Additional room lighting was manually switched on at ~ 08:00 and off at ~ 22:00. The black gobies in the communal tanks also received natural light from windows. All adult fish were fed frozen thawed and finely chopped Mysis shrimp, Euphasia shrimp, Pandalus shrimp, blue mussels and Alaskan pollock once per day to apparent satiation.

### Spawning

Spawning aquaria for sticklebacks and black gobies were set up in a dedicated room. These aquaria received flow-through of the same filtered and temperature-controlled seawater as described above. The photoperiod and feeding regime were the same as for the holding tanks (described above).

Starting on 13 June, male sticklebacks showing breeding colouration (red throat and blue eye colour; Frischknecht, 1993) were placed in spawning aquaria (12 aquaria, 19 L, 36 × 25 × 30 cm [L × W × H], water level 24 cm) equipped with sand as bottom substrate, an artificial plant and plant material for nest construction (collected from a nearby bay, filamentous green algae *Cladophora* sp. and *Ascophyllum nodosum* seaweed, following Candolin, 1997). One male was placed into each tank and allowed to build a nest, whereafter they were each provided with a gravid female (starting on 16 June, introduced in the morning, ~ 09:00). Females were removed once successful mating was confirmed (male chasing the female away, female visually emptied of eggs). If no mating had occurred by the evening (~18:00), the females were removed and a new, gravid, female was provided to unmated males the following day. Males that were not building a nest and/or that did not court the female were exchanged for new males. Three spawnings occurred intermittently on 17, 18 and 20 June (Supplementary Material, Table S1). Mated males were left undisturbed to care for the eggs. During the mating and nest caring period, males were fed once per day with newly hatched artemia and frozen mysis shrimp.

Ten pairs (one male and one female per aquarium) of reproductively mature adult black gobies were sorted into glass aquaria (31 or 46 L, 50 × 25 × 25 or 55 × 30 × 28 cm [L × W × H]) on 15 June. Aquaria were equipped with mixed sand and gravel bottom substrate, one larger rock, bladderwrack algae and a PVC pipe with mesh covering one end and lined with an acetate sheet. Six spawnings occurred on 18 June (one pair), 20 June (two pairs, one continued into 21 June), 25 June (one pair), 27 June (one pair) and 28 June (one pair) (Supplementary Material, Table S1). Once spawning was complete, the female was removed and the male was left to care for the eggs. Eggs and their male parent were also collected from the large holding tanks from five opportunistic spawning events (two on 15 June, the male parent could not be identified for one brood and eggs consequently did not survive; two on 17 June; one on 21 June; Supplementary Material, Table S1), as described above, which were set up in the black goby holding room. In these tanks, both salinity and temperature was naturally fluctuating (28.1 ± 0.67 PSU; temperature, 14.9 ± 0.39°C, daily average data from the continuous monitoring system at the research station, 15–21 June 2022: http://www.weather.loven.gu.se/kristineberg/en/data.shtml).

### CT_max_ methods

#### Embryonic CT_max_

**Figure 1:**
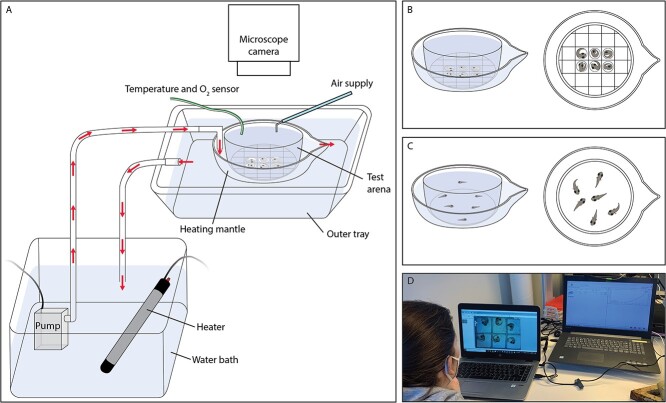
Schematic illustration of the embryo and larval CT_max_ setup. **(A)** Complete setup showing the test arena (a glass dish, also depicted in B and C), from which embryo and larval behaviour was observed and recorded (via a microscope camera for embryos). The test arena was supplied with air bubbling through a modified hypodermic needle, and was equipped with a temperature and O_2_ sensor. The test arena dish was placed in a larger glass bowl, which formed a flow-through heating mantle for temperature ramping. Water was pumped from a water bath equipped with heaters and flowed in on one side of the heating mantle, a lip allowed the water to flow out from the heating mantle on the other side, into an outer tray and then back to the water bath; arrows indicate water flow direction. **(B)** Test arena within the larger, glass, flow-through heating mantle, with a mesh fixed to the bottom for embryo separation. **(C)** Test arena within the larger, glass, flow-through heating mantle, with freely moving larvae. **(D)** Embryo movements, temperature and O_2_ are monitored on computers via a microscope camera and the temperature and O_2_ sensor.

Embryo CT_max_ trials were conducted in a 30 mL glass dish (the test arena), with a mesh fixed to the bottom (Fig. 1A, B)—mesh size was adapted to the size of the embryos being tested. The test arena was filled with 25 mL of surface water and was supplied with air bubbling through a blunted hypodermic needle. The test arena was placed in a larger glass bowl (140 mL), which formed a flow-through heating mantle, with water pumped in (via an Eheim Universal 300, Germany pump) from a water bath on one side of the bowl, and a lip allowing the water to flow out from the bowl on the other side into a surrounding tray, which had an outflow back into the water bath (Fig. 1A). The test arena walls were elevated above the glass bowl so that no water from the flow-through heating mantle entered the area where the embryos were placed. Water temperature in the test arena was adjusted by heaters in the water bath, with a heating rate of 0.30 ± 0.01 and 0.26 ± 0.01°C per min (mean ± se) for sticklebacks and black gobies, respectively (Supplementary Material, Fig. S1). Heating rate was recorded and monitored during the trials and water was added or removed from the water bath to ensure a steady heating rate. Temperature and water oxygen content was continuously recorded using a robust fiber-optic oxygen sensor (OXROB-10) and a temperature sensor (PT100) connected to an oxygen and temperature meter (FireSting-PRO, Pyro Science, Aachen, Germany).

Stickleback and black goby embryos were collected 5–6 and 4–8 days post-fertilization (dpf) (90–108 and 76–99 degree-days), respectively (see Supplementary Material, Fig. S6 for relationship between days post-fertilization and CT_max_), and placed in the cells of the mesh in the test arena (Fig. 1B) using a plastic pipette. The embryos were left here at holding temperature (see Supplementary Material, Fig. S5 for relationship between holding tank temperature and CT_max_) for approximately five minutes (habituation; Supplementary Material, Fig. S1). We ran six stickleback and 6 to 13 black goby embryos per trial, however if an embryo hatched during a trial, this was noted, and the recorded movement data were subsequently excluded for that individual, leaving two to six (all embryos from stickleback trial 5 hatched) and 4 to 13 embryos successfully tested per trial for sticklebacks and black gobies, respectively. Trials consisted of embryos from the same clutch (see Supplementary Material, Fig. S4 for relationship between male parent identity and CT_max_). After the period of habituation to the setup, the heater was turned on in the water bath and embryonic behavioural response to heating was recorded by an observer as number of individual movements over 30 second intervals at the start of every minute during the trials (i.e. 30 seconds recording, 30 seconds not recording). Embryo movement was observed via a microscope camera (Leica M205 c fitted with Axiocam 305 color, viewed through Zen lite 3.1. for the smaller black goby embryos, and Leica Wild M3Z fitted with a DinoEye camera, viewed through DinoCapture 2.0 for the larger stickleback embryos) connected to a computer (Fig. 1). The presence of an individual’s heartbeat was also recorded whenever the embryo position allowed a clear view of the heart. The endpoint for CT_max_ was defined as the temperature (recorded at the start) of the 30 second observation period, during which each individual embryo’s last movement occurred. Once the CT_max_ was reached in the entire testing group, embryos were continually visually checked for heartbeats and when no heartbeats were detected among any individuals in the group, embryos were removed from the test chamber and placed in ~ 20°C water to recover. Survival rate was initially checked after 30–60 min and was 96% for black gobies (for 9/10 trials; no 30–60 min check for Trial 1) and 95% for sticklebacks (7 trials). A second check was conducted at 24 h and was 84% for black gobies (for 9/10 trials; Trial 2 was checked only at 7 h [64% survival]) and 78% for sticklebacks (for 6/7 trials; Trial 3 was checked only at 12 h [100% survival] and 36 h [50% survival]). Note that for black gobies, there were additional embryos in several trials, other than those for which behavioural observations were recorded, and it was not possible to track the individuals during survival, so survival rate was estimated for the total observed and unobserved embryos. Additionally, approximately 16% of black gobies and 27% of sticklebacks were observed to hatch during the survival monitoring period after the CT_max_ trials.

#### Larvae CT_max_

Acute thermal tolerance was measured in groups of larvae (7–11 per group for sticklebacks; 6–10 per group for black gobies) that were collected within 36 h of hatching (8–9 dpf for sticklebacks; 4–12 dpf for black gobies; see Supplementary Material, Fig. S6 for relationship between days post-fertilization and CT_max_). Trials consisted of larvae from the same cohort (i.e. siblings) (see Supplementary Material, Fig. S4 for relationship between male parent identity and CT_max_). The CT_max_ setup was the same as for embryos, except that larvae were placed in a 40 mL test arena (Fig. 1C), without a mesh fixed to the bottom. Larvae were added to the test arena at holding temperature (see Supplementary Material, Fig. S5 for relationship between holding tank temperature and CT_max_) and after a habituation period (6.5 ± 2.8 min, mean ± S.D.), the heater in the water bath was switched on (heating rate of 0.28 ± 0.01 and 0.26 ± 0.01°C per min, mean ± se for sticklebacks and black gobies, respectively; Supplementary Material, Fig. S2).

Following Andreassen *et al.* (2022), larvae were continuously visually monitored and the temperature at which individual larvae failed to respond to five consecutive touches (using a dissection probe modified with 2 mm plastic cannula tubing to make a flexible and blunt end) at 3 sec intervals was defined as their CT_max_. Upon reaching their CT_max_, larvae were removed from the test chamber and placed in ~ 20°C water to recover. Recovery and survival were monitored over the following 24 h. For black gobies, the survival rate was 55% (43 alive, 35 dead/non-responsive) after 30 min and 48% after 24 h (56 larvae; no 24 h survival recorded for Trial 3, 5, 6). For sticklebacks the survival rate was 100% after both 30 min (*n* = 54; no 30 min survival check for Trial 5) and 24 h (*n* = 33; survival was only recorded at 48 h for Trial 5 [33% survival] and 3 h for Trial 6 [100% survival]; no 24 h check for Trial 7).

#### Adult CT_max_

Acute thermal tolerance was measured in groups of adult fish (7–10 per group for sticklebacks; 6–10 per group for black gobies), in one of two test arenas, following an established protocol (Morgan *et al.,* 2018). The first, larger, setup consisted of a Styrofoam CT_max_ arena (50 × 32 × 32 cm [L × W × H], 17.5 cm water depth) connected to a Styrofoam water bath (37 × 37 × 34.5 cm [L × W × H], 6.5 cm water depth) (total volume of 35 L in system), with three water pumps (Eheim Universal 300, Germany) all placed inside the water bath, two of which transferred water from the water bath to opposite corners of the CT_max_ arena (one had a valve to adjust flow) and one which pumped water out of the arena to the water bath, from an outlet in the bottom; outlets were covered with mesh. Heating was via a 500 W and 300 W heater in the water bath. The second, smaller setup consisted of a single heating tank (25 × 20 × 18 cm [L × W × H], filled with 12 L water), divided by a mesh into a heating compartment and a fish compartment (Morgan *et al.,* 2018). The heating compartment contained a custom-made cylindrical steel heating case, consisting of an inflow nipple, a wide outflow and a 300 W coil heater and a water pump (Eheim Universal 300, Germany).

Groups of adult fish were left in the CT_max_ arenas at holding temperature for 30 min (habituation period) (see Supplementary Material, Fig. S5 for relationship between holding tank temperature and CT_max_) before the heaters were switched on. The heating rate was 0.28 ± 0.02 and 0.27 ± 0.01°C per min (mean ± S.E.) for sticklebacks and black gobies, respectively (Supplementary Material, Fig. S3), with temperature measurements manually recorded via a Testo thermometer (testo-112, Testo, Lenzkirch, Germany) inside the CT_max_ arena. We defined CT_max_ as the temperature at which individuals experienced loss of equilibrium for 3 sec. For black gobies, survival rate was 94% after 30 min after the CT_max_ trials. For sticklebacks, survival rate was 97% after 0.5–2 h (Trials 1–4 checked after 30 min [100% survival]; Trials 5–7 checked after 1.5–2 h [95% survival]).

### Data analysis

Analyses and visualizations were performed in RStudio, version 2022.7.2.76 (RStudio Team, 2022) (R, version 4.2.2; R Core Team, 2022). Linear mixed-effects models were fitted with the packages *lme4* (Bates *et al.,* 2015), *lmerTest* (Kuznetsova *et al.,* 2017) and *rstatix* (Kassambara, 2022) to compare levels of the fixed effects. Marginal and conditional R^2^ were calculated with the *MuMIn* package (Bartoń, 2022). Model assumptions were visually assessed using residual plots, as well as tested using the *DHARMa* package (Hartig, 2020). The significance of effects was considered at the significance level *α* = 0.05. Prior to analysis, seven black goby larvae, which had a note that something went wrong during the trial that affected measurement of their CT_max_, and one black goby embryo, which did not move for the duration of the trial, were excluded from the dataset.

The relationship between last movement and last heartbeat in embryos was analysed with a linear mixed-effects model with temperature (°C) as the response variable, a categorical fixed effect of endpoint type (last movement or last heartbeat) and embryo identity as a random effect (Supplementary Material, Table S2, S3). A separate model was run for each species.

A linear mixed-effects model was also used to compare CT_max_ across life stages. The model included CT_max_ temperature (°C) as the response variable, life stage as a categorical fixed effect and a unique trial identifier as a random effect (Supplementary Material, Table S4, S5). CT_max_ for embryos was the temperature of the last movement. A separate model was run for each species. For black gobies, seven individuals (all embryos) were identified as outliers, with three identified as extreme outliers (defined as values <Q1–3*IQR or > Q3 + 3*IQR; all extreme outliers had CT_max_ values that were < 25°C), which were excluded from the analysis (see Supplementary Material, Table S5 for outputs of models with and without the extreme outliers).

## Results

**Figure 2:**
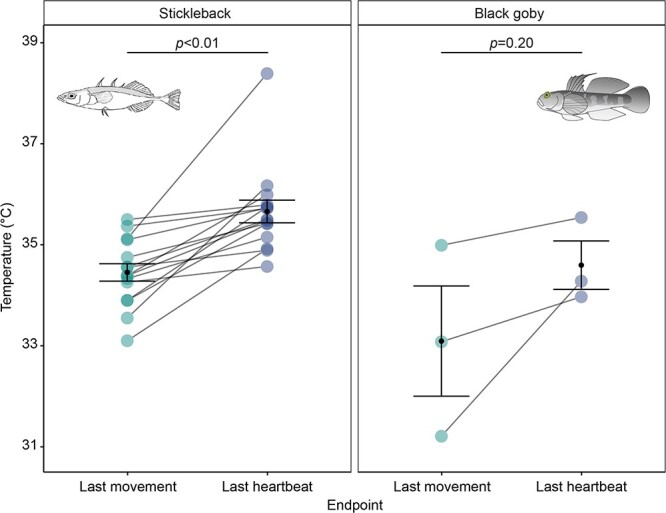
Temperature of the last movement (CT_max_) and last heartbeat of individual embryos, where data were available for both, in stickleback (left panel) and black goby (right panel). Lines between last movement and last heartbeat connect data from the same individual. Black points and error bars show the mean ± S.E. for the last movement and last heartbeat, respectively. Statistical results comparing differences between last movement and last heartbeat (within each species) are denoted with lines and p-values in the figure.

For individual embryos in which it was possible to detect when the heart stopped beating (stickleback, *n* = 15; black goby, *n* = 3), there was a significant difference between the temperature of the last movement (34.45 ± 0.17°C [mean ± S.E.]) and the temperature of last heartbeat (35.66 ± 0.22°C [mean ± S.E.]) for sticklebacks, with the last movement preceding the last heartbeat by 1.21°C on average (linear mixed-effects model, *β*±S.E. = 1.21 ± 0.23, t_(14)_ = 5.21, *P* < 0.01, *R_2_* = 0.39; Fig. 2, Supplementary Material, Table S2). For black gobies, the temperature of the last movement (33.09 ± 1.09°C [mean ± S.E.]) preceded the temperature of last heartbeat (34.60 ± 0.48°C [mean ± S.E.]) by 1.50°C on average, however this was not statistically significant (linear mixed-effects model, *β±*S.E. = 1.50 ± 0.79, t_(2)_ = 1.90, *P* = 0.20, *R_2_* = 0.24; Fig. 2, Supplementary Material, Table S3).

**Figure 3:**
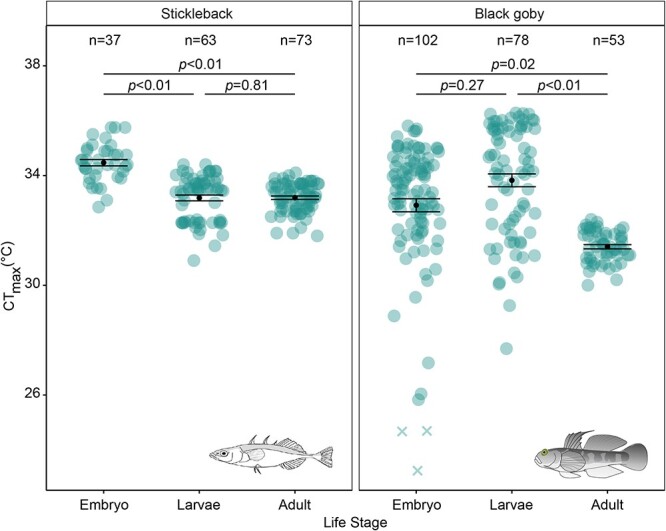
CT_max_ across life stages (embryo, larvae, adults) in stickleback (left panel) and black goby (right panel). Data points represent individual fish’s CT_max_; black points and error bars show the mean CT_max_ ± S.E. for each life stage. Sample size for each species and life stage is given in the figure. Crosses identify extreme outliers (<Q1–3xIQR), that were excluded before analysis. Statistical results comparing differences in CT_max_ between life stages (within each species) are denoted with lines and p-values in the figure.

For sticklebacks, there was no significant difference between the CT_max_ of larvae (33.18 ± 0.10°C [mean ± S.E.], *n* = 63) and adults (33.19 ± 0.06°C [mean ± S.E.], *n* = 73) (linear mixed-effects model, *β*= − 0.07, S.E. = 0.29, df = 18.87, t = −0.25, *P* = 0.81); however, there was a significant difference between the CT_max_ of embryos (34.47 ± 0.12°C [mean ± S.E.], *n* = 37) compared with both larvae (linear mixed-effects model, *β*= − 1.19, S.E. = 0.30, df = 19.91, t = −3.90, *P* < 0.01) and adults (linear mixed-effects model, *β*= − 1.26, S.E. = 0.30, df = 19.97, t = −4.27, *P* < 0.01) (Fig. 3; Supplementary Material, Table S4). For black gobies, there was no significant difference between the CT_max_ of embryos (33.18 ± 0.19°C, *n* = 99 [without 3 extreme outliers]) and larvae (33.83 ± 0.23°C [mean ± S.E.], *n* = 78) (linear mixed-effects model, *β*=0.64, S.E. = 0.57, df = 21.49, t = 1.13, *P* = 0.27); however, there was a significant difference between the CT_max_ of both embryos (linear mixed-effects model, *β*= − 1.61, S.E. = 0.64, df = 21.47, t = −2.52, *P* = 0.02) and larvae (linear mixed-effects model, *β*= − 2.25, S.E. = 0.65, df = 21.48, t = −3.46, *P* < 0.01) compared with adults (31.40 ± 0.08°C [mean ± S.E.], *n* = 53) (Fig. 3; Supplementary Material, Table S5).

**Figure 4:**
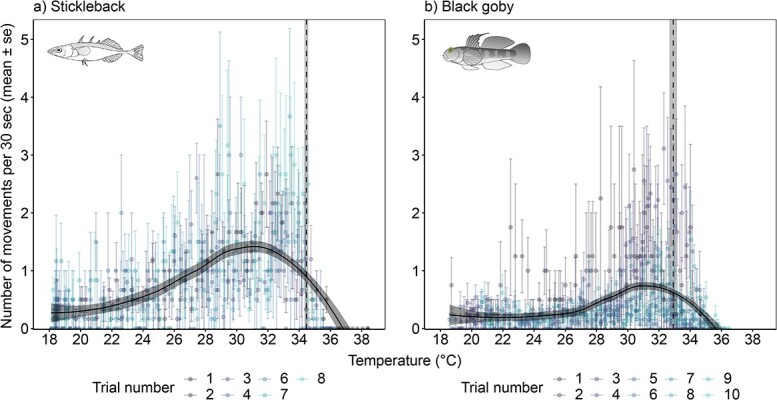
Number of embryo movements (mean ± S.E.) recorded during 30-sec observation periods during acute thermal ramping (7 trials for sticklebacks, 10 trials for black gobies). Movements of individual embryos were recorded during intervals of 30 sec (observation period) followed by 30 sec without observations for the duration of the trial; data points with error bars represent the mean ± S.E. for each trial (black goby, *n* = 4–13; stickleback, *n* = 2–6 individuals per trial) at each time period during thermal ramping (~0.3°C min^−1^; Supplementary Material, Fig. S1). Smoothed black line is fitted with a loess curve ± S.E. CT_max_ was taken as the last movement of the individual embryo. Vertical dashed lines and shaded bars show the mean CT_max_ ± S.E. of all embryos for each species.

Measurements of embryonic movement during thermal ramping showed that activity increased up until approximately 31°C for both species, whereafter it decreased, before the embryos stopped moving (Fig. 4). The CT_max_ temperature (temperature of the last movement) was 34.47 ± 0.12 and 32.92 ± 0.24°C (mean ± S.E.) for stickleback and black goby embryos, respectively (Fig. 4).

**Table 1:** Considerations and recommendations for conducting CT_max_ trials on fish embryos

**Experimental component**	**Recommendation**
CT_max_ arena	• Use a mesh appropriate to the size of the embryo to keep embryos from moving out of view during trials.
	• Use an air supply to maintain consistent oxygen levels during heating. Since water in the test arena is not exchanged, there is the potential for supersaturation during heating, or hypoxia from the metabolic demand of the embryos.
Ramping rate	• Establish repeatable rates that are suitable for the study species (e.g., 0.3°C per min).
Reduce experimenter bias	• The individual recording behavioural observations should not be aware of the temperature.
**Consideration**	**Recommendation**
Developmental stage of embryos, embryogenesis	• Report developmental stage of embryos; thermal tolerance can change during embryogenesis and measurement of spontaneous activity and heart rate is more suitable at later developmental stages.
Premature hatching	• Pilot tests are needed to check optimal time points for testing each species.
Holding temperature	• Standardize and control egg incubation temperatures as CT_max_ is sensitive to developmental and acclimation effects of holding temperatures.• Report degree-days.
Small eggs may be difficult to observe	• Microscope camera and software for observing and recording may make any size possible.
Survival	• Survival of embryos should be monitored for 24 h following the CT_max_ trial.
Automated tracking of embryos	• Future work should aim at automating tracking of embryo movements and heart rates. This allows continuous observation, which provides higher precision.

All embryos in a trial continued to be heated until no movements and no heartbeats were observed in any individuals in the trial. For black gobies, this resulted in 2.16 ± 0.22°C (mean ± S.E.)/1.52°C (median) and a maximum of 11.04°C of additional warming after reaching CT_max_. For sticklebacks, the equivalent was 1.56 ± 0.18°C (mean ± S.E.)/1.23°C (median) and a maximum of 4.87°C.

## Discussion

Our novel method of measuring CT_max_ in fish embryos, where CT_max_ was defined by the temperature at which the embryos stopped moving, was successful and practical. In addition, this method provided temperatures of acute thermal tolerance limits that are comparable with CT_max_ temperatures in larvae and adults. Specifically, we show that measurements based on cessation of movement in stickleback embryos occur at lower temperatures than when measuring last heartbeat (which has been proposed as a method for measuring CT_max_ in embryos), suggesting that recordings of last movement are more comparable with measurements of CT_max_ based on LOE in other life stages. A similar trend was observed in black gobies despite a very low number of individuals in which the two methods could be directly compared. Our embryonic movement CT_max_ method thus provides a useful tool for studies of acute thermal tolerance in embryos and across life stages.

Since LOE can be difficult to measure in fish embryos, previous experiments have instead typically used the temperature at which the heart stops beating to define CT_max_. For example, in an experiment on Chinook salmon *Oncorhynchus tshawytscha*, Del Rio *et al.* (2019) defined CT_max_ as the temperature at which the heart stopped beating for 30 sec, while Zebral *et al.* (2018) defined CT_max_ in annual killifish *Austrolebias nigrofasciatus* as the temperature at which the heart stopped beating for 5 sec. While this method might be useful, it comes with the drawback that it cannot be compared with CT_max_ in other life stages, since the heart often does not stop beating when LOE is reached in juveniles and adults (Angilletta *et al.,* 2013; Ekström *et al.,* 2016) and can continue up to the point of rigor (Lutterschmidt and Hutchison, 1997). This means that the use of cessation of heartbeat can lead to an overestimation of CT_max_ in embryos, making comparisons of heartbeat measurements with movement-based measurements of CT_max_ in larval, juvenile and adult life stages unreliable (Del Rio *et al.,* 2019). Indeed, in this study, we observed a CT_max_ (based on loss of movement) in stickleback embryos that was significantly higher than that recorded for larvae and adults; this difference would have been even greater if using loss of heartbeat as the measured endpoint. For black goby embryos, a similar trend was observed despite a very low number of individuals in which the two methods could be directly compared. The lower sample size for this specific comparison was due to the small size of black goby embryos (egg size: 2.14 x 0.64 mm, LxW [Borges *et al.,* 2011]), which made it difficult to detect the heartbeat, with the last heartbeat observed in only 3% of individuals. This can be compared with the larger stickleback embryos (egg size: 1.33–2.16 mm, D [Glippa *et al.,* 2017]), in which the final heartbeat could be detected in 41% of the individuals. Our results thus show that the novel method of measuring CT_max_ based on loss of movement rather than loss of heartbeat in fish embryos is a more easily applicable method across a broader size range of embryos. In addition, this method provides a more conservative measure of CT_max_ that is appropriate to use when comparing differences between life stages. It should be noted that differences between life stages could of course be due to actual differences in acute thermal tolerance, but using different endpoints can result in erroneous results.

Our finding that embryos exhibited a period of increased activity, which peaked at approximately 2–3°C before CT_max_, is in line with the theory that ectotherms may exhibit behavioural strategies to avoid physiological damage that occurs near their CT_max_ (Lutterschmidt and Hutchison, 1997; Kochhann *et al.,* 2021). These behavioural strategies may be associated with seeking local thermal refugia or alternative habitats (Kochhann *et al.,* 2021). Kochhann *et al.* (2021) describe the temperature at which increased activity occurs as the agitation temperature (T_ag_). With continued increase in warming, a period of inactivity follows, during which CT_max_ occurs (Lutterschmidt and Hutchison, 1997; Kochhann *et al.,* 2021), which was also found here. Our results are in line with other quantitative studies, for example an increase in activity was observed 6°C before CT_max_ in the neotropical cichlid *Cichlasoma paranaense* (Brandão *et al.,* 2018). In adult Amazonian cichlids *Apistogramma agassizii* and *Mesonauta insignis*, T_ag_ was observed at 4 and 5.4°C, respectively, prior to CT_max_ (Kochhann *et al.,* 2021). It is worth noting that all embryos in a trial in our study continued to be heated until no movements and no heartbeats were observed in any individuals in the trial. For black gobies, this resulted in a maximum of 11.04°C of additional warming after reaching CT_max_ (this individual was notably still alive 30 min after the CT_max_ trial; individual survival was not tracked after 24 h). For sticklebacks, the equivalent was up to 4.87°C above the last recorded movement (this individual was alive after 1 h but had died by 24 h post trial). Despite these sometimes-high additional warming temperatures, survival rates were very high 30–60 min post-trial (96% survival for black gobies and 95% for sticklebacks), and relatively high 24 h post-trial (84% survival for black gobies and 69% for sticklebacks). Furthermore, approximately 16% of the black goby embryos and 27% of sticklebacks hatched within 24 h after the CT_max_ trials. This suggests a surprising robustness of the embryonic life stage to acute warming, which might be explained by an adaptation to tolerate short term heating in these demersal spawning, shallow water fish.

While the novel method for measuring acute upper thermal tolerance limits in fish embryos presented here is promising, several aspects must be considered in order to obtain reliable results (Table 1). Some of these concerns are valid in any experiment where CT_max_ is measured, and some are more specific to embryonic CT_max_ measurements. For example, heating rate during trials has been found to affect CT_max_ (Åsheim *et al.,* 2020), and it is therefore important to use the same heating rate across life stages (or species or treatment groups) if the purpose is to compare thermal tolerance limits. Ramping rates during trials should allow an individual’s internal temperature to track that of its surrounding environment, and represent natural conditions (Terblanche *et al.,* 2011; Vinagre *et al.,* 2015; Morgan *et al.,* 2018). Ramping rates that are too fast could lead to an overestimation of upper thermal tolerance limits due to a difference between internal and external temperature, especially in larger organisms. On the other hand, slower ramping rates could allow individuals to develop thermal tolerance due to acclimation and longer exposure to heat stress, leading to underestimation of CT_max_ (Terblanche *et al.,* 2007; Kingsolver and Umbanhowar, 2018; Ern *et al.,* 2023). Ramping rates used during CT_max_ trials are generally faster than those naturally observed (although similar heating rates can be experienced by fish in the intertidal zone, during extreme upwelling events, or when moving through a thermocline [Bates and Morley, 2020; Genin *et al.,* 2020; Ern *et al.,* 2023]), however, their ecological relevance has been supported as CT_max_ is correlated with both tolerance to slower warming rates and to the natural upper temperature range of ectotherms (Sunday *et al.,* 2012; Åsheim *et al.,* 2020; Desforges *et al.,* 2023). However, to allow for comparisons using our methods (across species and life stages), we encourage the usage of equal ramping rates across all life stages and throughout the duration of the trial, especially if activity is being monitored.

The developmental stage of the tested embryos is another consideration for researchers applying the method presented here. We used spontaneous activity and heart rates as indicators of thermal tolerance, but this may be difficult to measure in early stages of embryogenesis. Thus, our method is, in general, restricted to embryos in later stages of development (i.e. segmentation stage and beyond). This is an important consideration as some literature suggests that thermal tolerance is lower during early embryogenesis compared with later in embryogenesis in some species (Rombough, 1997; Bunn *et al.,* 2000; Del Rio *et al.,* 2019). As a result, our method may overestimate thermal tolerance in species where early embryogenesis represents a thermally critical phase. We suggest that researchers should report the embryonic stage measured as accurately as possible to facilitate comparisons across studies and species (Table 1).

When measuring CT_max_ in late-stage embryos, premature hatching can be an issue if the embryos are tested during the last days before natural hatching. Pilot tests are needed to ensure that the embryos are tested at an optimal time point, which could differ between species (Table 1). The incubation time is likely to be affected by (holding) temperature in many species, so egg incubation temperatures should be controlled and standardized. Egg size is another important aspect, since smaller eggs can be more challenging to work with compared with species that have larger eggs. The methods, setup and microscope used to observe the embryos should be modified to the egg size of the studied species. Movements in the setup should be minimized to obtain videos with a quality suitable for quantification of spontaneous movements in the embryos. High air bubbling will both disturb the water surface and can cool down the water. We recommend monitoring oxygen levels to maintain stable levels throughout the heat ramping, while also minimizing the amount of air bubbling required.

## Conclusions

The novel method for measuring acute upper thermal tolerance limits in fish embryos based on last movements is a high throughput method giving similar and comparable CT_max_ temperatures as for larvae and adults. While not tested here, the method should also work well for other taxa. Measurements of CT_max_ based on last movement are more conservative than cessation of heartbeats, easier to record, and work well with both large and small embryos during later developmental stages. The method described here hence allows for comparisons of acute thermal tolerance of embryos between species, across life stages within species, and as a response variable to treatments. Although there are limitations to using CT_max_ in embryos as a determination of the vulnerability of species to climate change, developing techniques to measure and compare CT_max_ across life stages remains important from a conservation perspective. We encourage researchers to apply the method presented here to additional species, including those with long embryogenesis, in order to test the generality of the method, and with the ultimate goal of improving the method for more accurate usage across stages of embryonic and larval development and species.

## Supplementary Material

Web_Material_coad061
