# Supplementary material for: A novel method for measuring acute thermal tolerance in fish embryos
Source: Conserv Physiol. 2023 Aug 8;11(1):coad061. doi: 10.1093/conphys/coad061 (PMC10410291; doi:10.1093/conphys/coad061)
Supplement: Web_Material_coad061 [file web_material_coad061.pdf]

Supplementary material

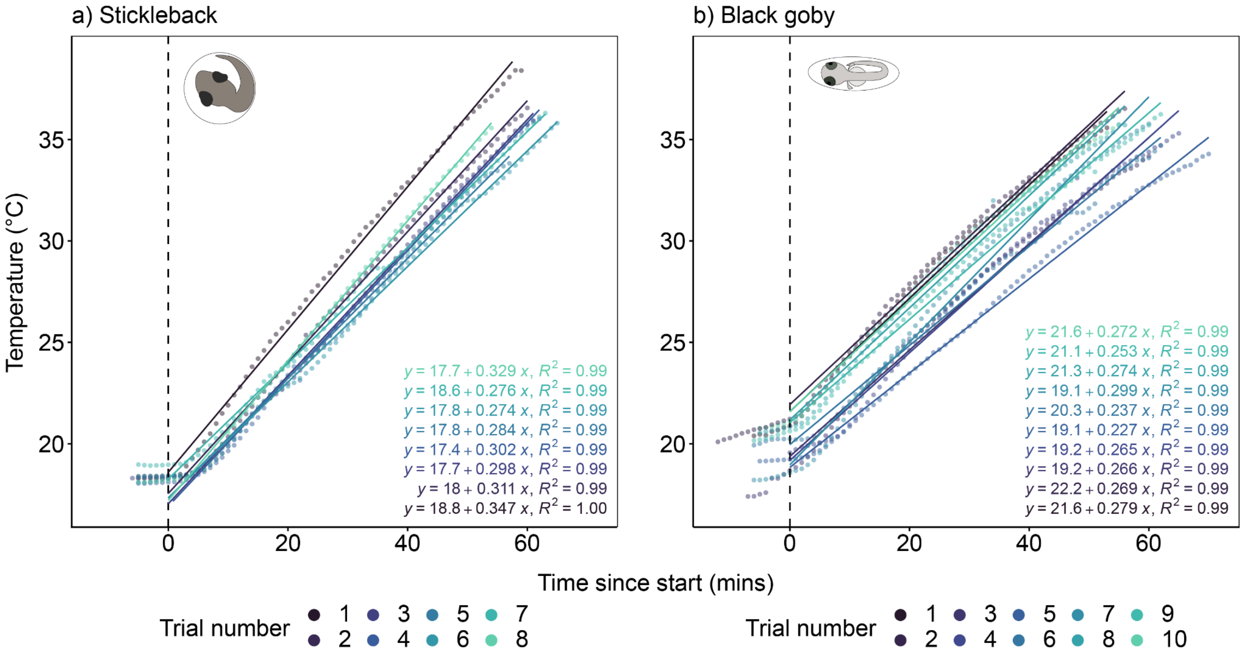

Figure S1. Temperature ramping during embryo CT_{max} trials. Fit with linear regression line. Vertical dashed line indicates when the heater was switched on.

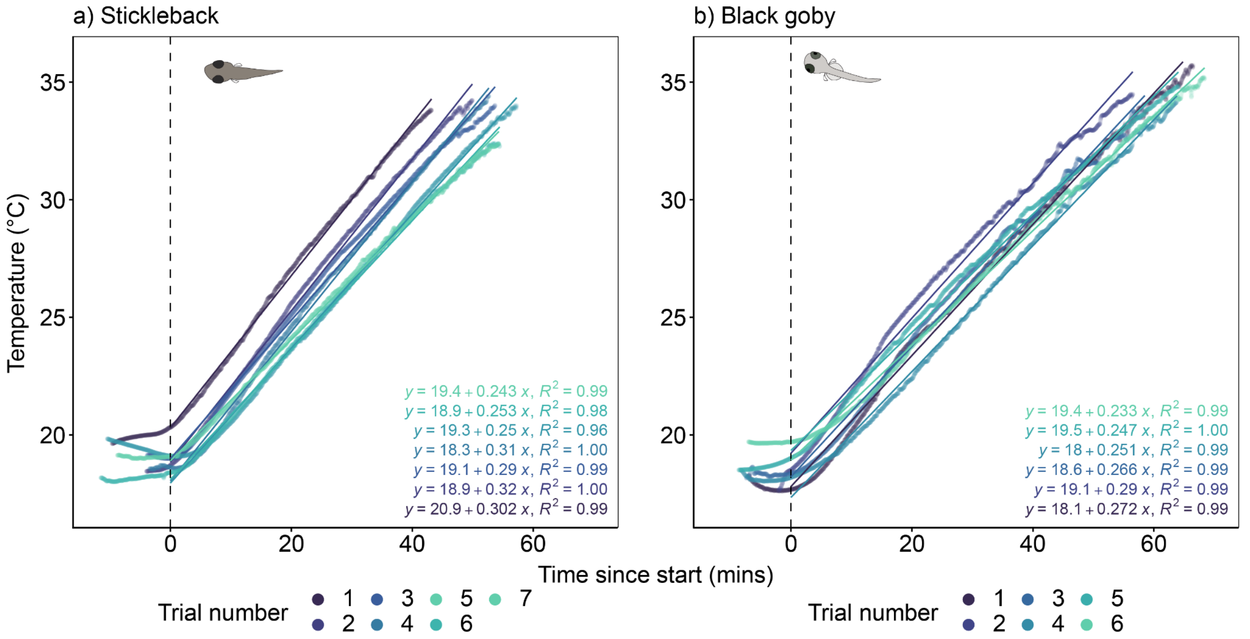

Figure S2. Temperature ramping during larval CT_{max} trials. Raw data points from PyroScience log, fit with linear regression line. Vertical dashed line indicates when the heater was switched on. No temperature data were logged for black goby trials 7-9.

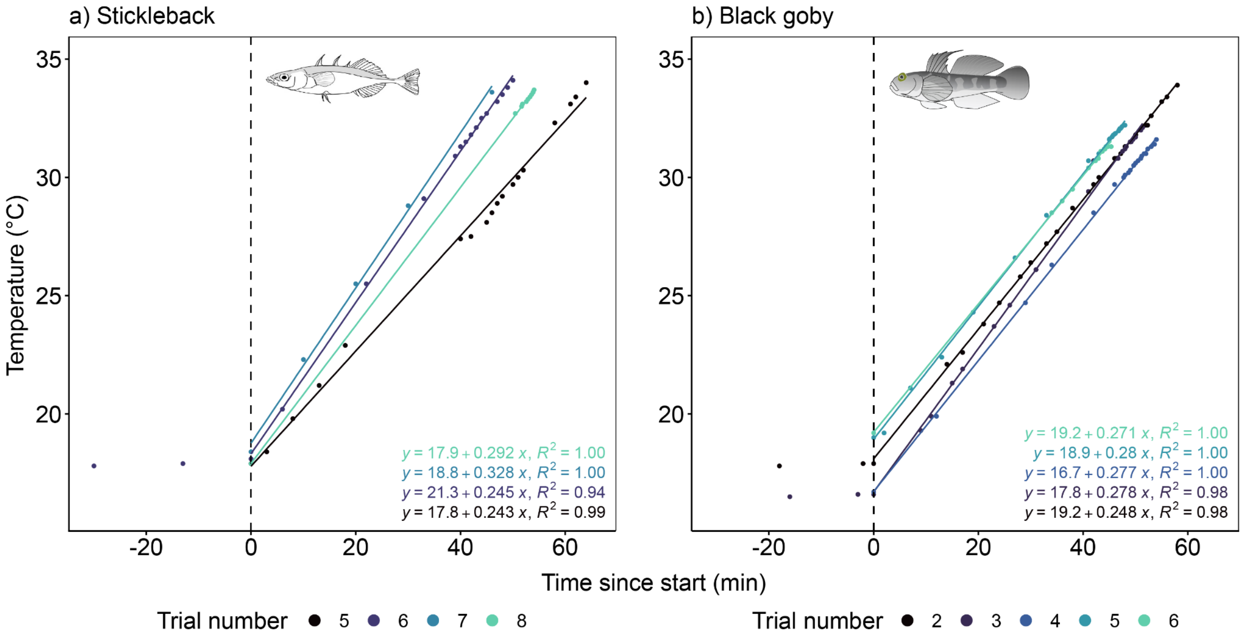

Figure S3. Temperature ramping during adult CT_{max} trials. Temperature measurements were manually recorded via a Testo thermometer inside the CT_{max} arena. Fit with linear regression. Vertical dashed line indicates when the heater was switched on. Stickleback Trial 5 had a power outage at 11:05 (35 mins).

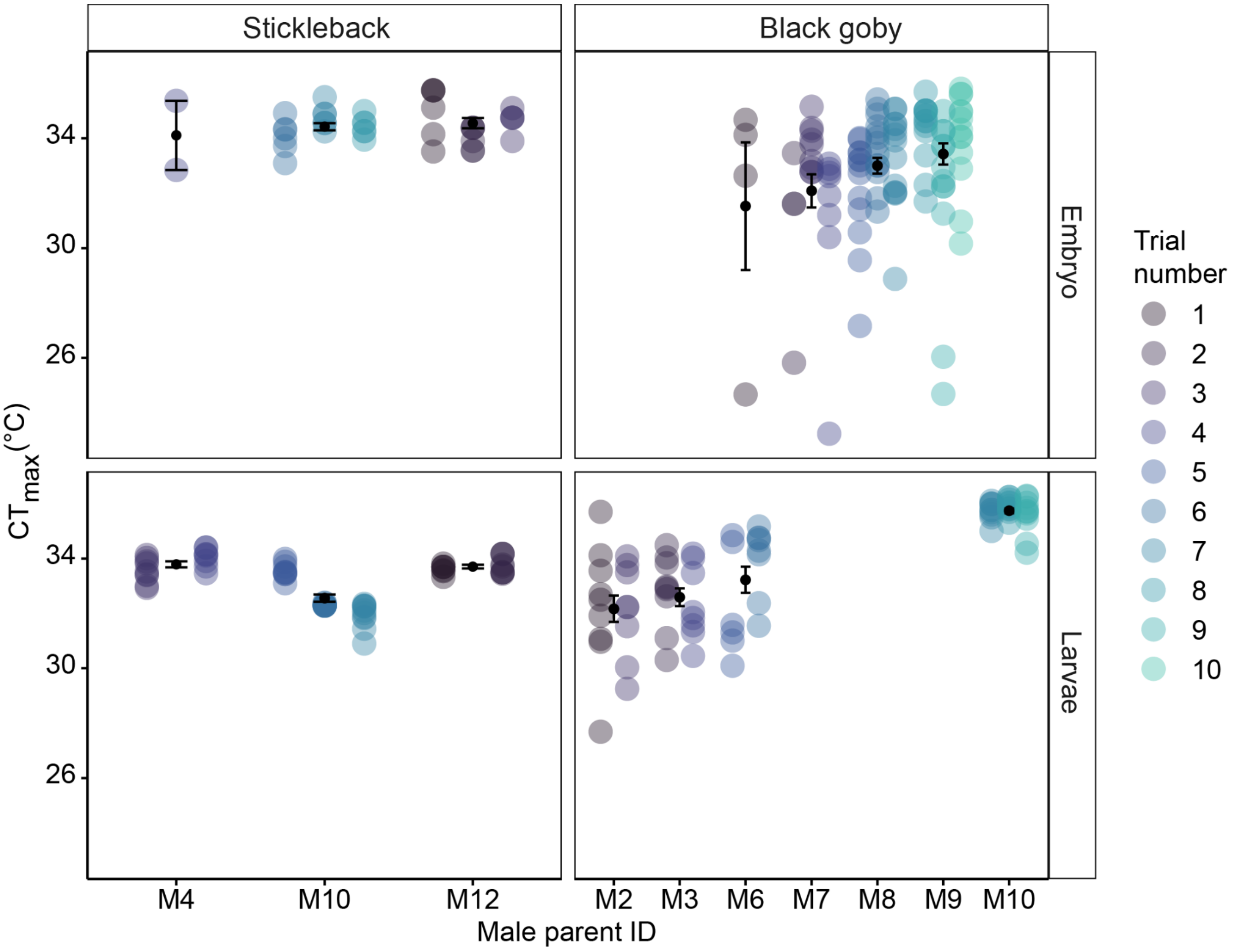

Figure S4. Relationship between CT_{max} and male parent identity. Black points and whiskers show mean ± S.E. of CT_{max}, grouped by male parent identity. Position of points within a male parent is separated by trial number.

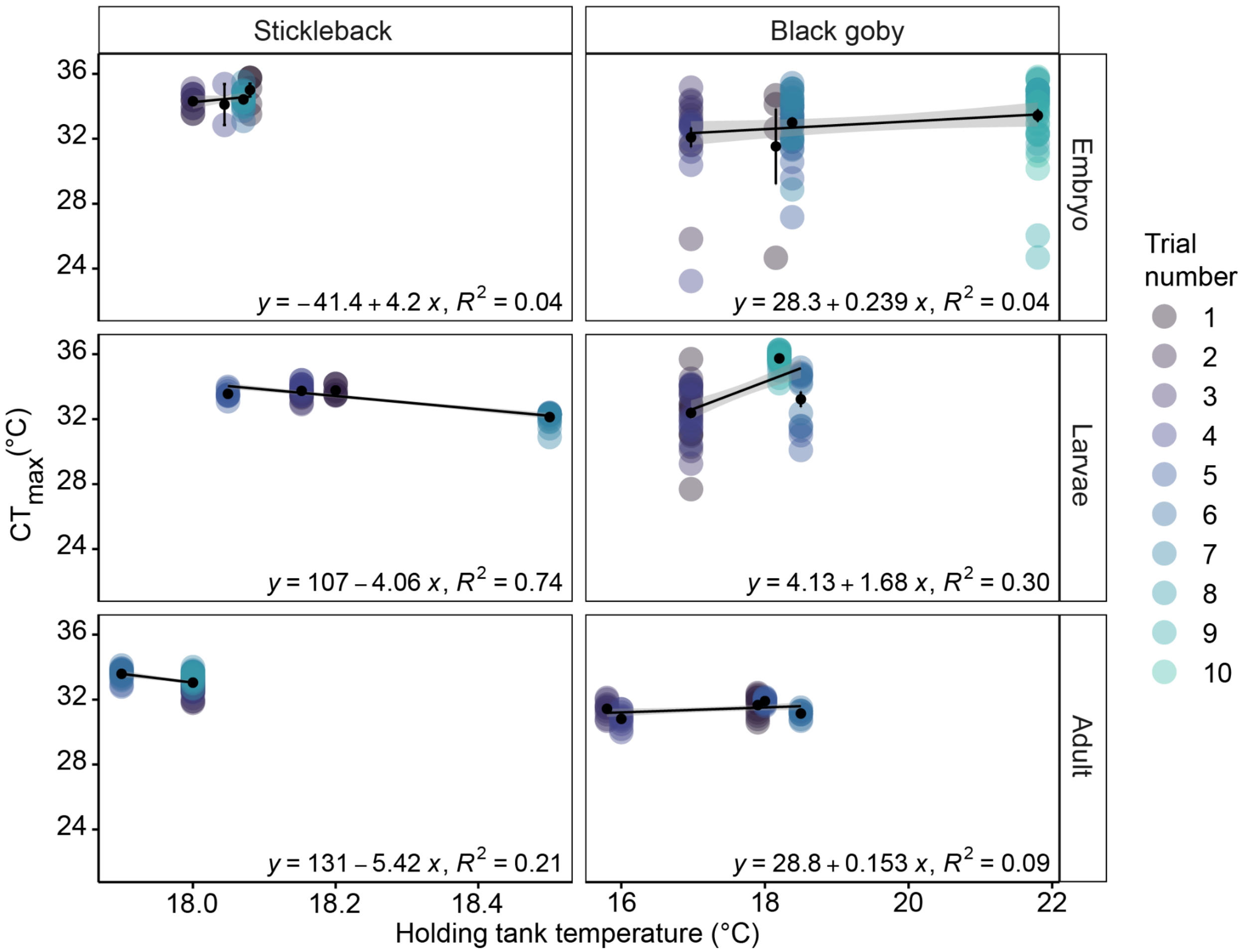

Figure S5. Relationship between holding tank temperature and CT_{max}. Black points and whiskers show mean ± S.E., grouped by holding tank temperature.

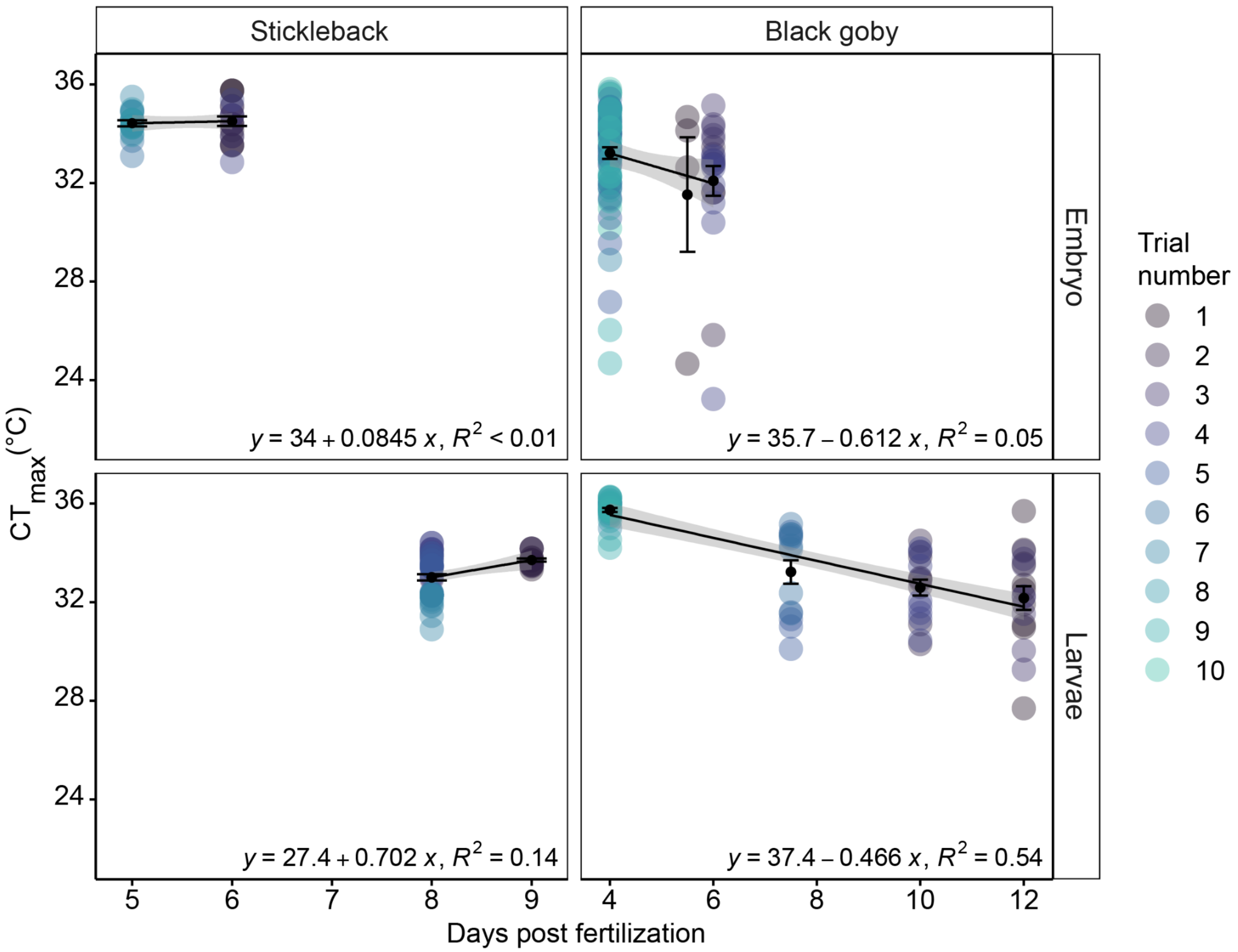

Figure S6. Relationship between days post fertilisation and CT_{max}. Black points and whiskers show mean ± S.E., grouped by days post fertilisation.

Table S1. Spawning dates and measurements for male three-spined sticklebacks and male black gobies guarding broods used in CT_{max} tests. (*) All embryos hatched during the trial. T = Trial.

Species	Male ID	Date spawned	Standard length, mm	Total length, mm	Mass, g	Used in which trials
G. aculeatus	4	18 June	46.40	52.09	1.06	Embryo (T4–5*), Larvae (T3–4)
G. aculeatus	10	20 June	39.18	45.04	0.688	Embryo (T6–8), Larvae (T5–7)
G. aculeatus	12	17 June	52.79	61.09	1.63	Embryo (T1–3), Larvae (T1–2)
G. niger	1 (no male)	15 June?	-	-	-	-
G. niger	2	15 June	81	98	9.3138	Larvae (T1,3)
G. niger	3	17 June	103	123	18.4896	Larvae (T2,4)
G. niger	4	18 June	103	126	22.5740	-
G. niger	5	20 June	100	121	20.1337	-
G. niger	6	20-21 June	82	101	10.3563	Embryo (T1), Larvae (T5–6)
G. niger	7	21 June	75	90	8.7700	Embryo (T2–4)
G. niger	8	25 June	91	112	14.7802	Embryo (T5–7)
G. niger	9	27 June	85	102	9.6433	Embryo (T8–10)
G. niger	10	28 June	95	116	15.5117	Larvae (T7–9)
G. niger	11	17 June	106	131	23.5235	-

Table S2. Model output for use of temperature of last movement vs last heartbeat as CT_{max} of sticklebacks (**Fig. 2**). The mixed-effects model includes fish identity as a random factor. Last movement is the intercept. Units are in °C.

Parameter	*Estimate, β*	*S.E.*	*df*	*t-value*	*p-value*
Intercept (Last movement)	34.45	0.20	25.26	172.02	**<0.01**
Endpoint (Last heartbeat)	1.21	0.23	14.00	5.21	**<0.01**
**Random effects**	σ^2	*S.D.*			
UniqueID	0.20	0.45			
Residual	0.40	0.64			
Observations	30				
N(UniqueID)	15				
Marginal R^2 / Conditional R^2	0.39/0.59				

Table S3. Model output for use of temperature of last movement vs last heartbeat as CT_{max} of black gobies (**Fig. 2**). The mixed-effects model includes fish identity as a random factor. Last movement is the intercept. Units are in °C.

Parameter	*Estimate, β*	*S.E.*	*df*	*t-value*	*p-value*
Intercept (Last movement)	33.09	0.84	3.04	39.26	**<0.01**
Endpoint (Last heartbeat)	1.50	0.79	2.00	1.90	**0.20**
**Random effects**	σ^2	*S.D.*			
UniqueID	1.20	1.09			
Residual	0.93	0.97			
Observations	6				
N(UniqueID)	3				
Marginal R^2 / Conditional R^2	0.24/0.67				

Table S4. Model output for CT_{max} across life stages for sticklebacks (**Fig. 3**). The mixed-effects model includes life stage (embryo, larva, adult) as a fixed effect and trial number as a random factor. Units are in °C. Larvae vs adult (sticklebacks) (β=-0.07, S.E.=0.29, df=18.87, t=-0.25, p=0.81)

Parameter	*Estimate, β*	*S.E.*	*df*	*t-value*	*p-value*
Intercept (Embryo)	34.45	0.22	20.96	157.78	**<0.01**
Larvae	-1.19	0.30	19.91	-3.90	**<0.01**
Adult	-1.26	0.30	19.97	-4.27	**<0.01**
**Random effects**	*σ^2*	*S.D.*			
UniqueTrial_no	0.30	0.54			
Residual	0.18	0.43			
Observations	173				
N(UniqueTrial_no)	22				
Marginal R^2 / Conditional R^2	0.35/0.75				

Table S5. Model output for CT_{max} across life stages for black goby (**Fig. 3**). The mixed-effects model includes life stage (embryo, larva, adult) as a fixed effect and trial number as a random factor. Units are in °C. Model 1 is a linear regression model including all fish, however seven embryos were identified as outliers (three extreme outliers [<Q1-3*IQR]). Model 2 excludes the three extreme outliers. Larvae vs adult (gobies) (β=-2.25, S.E.=0.65, df=21.48, t=-3.46, p<0.01)

	**Model 1**					**Model 2**				
Parameter	*Estimate, β*	*S.E.*	*df*	*t-value*	*p-value*	*Estimate, β*	*S.E.*	*df*	*t-value*	*p-value*
Intercept (Embryo)	32.69	0.42	21.29	78.76	**<0.01**	33.04	0.39	21.48	84.30	**<0.01**
Life stage (Larva)	1.00	0.60	21.60	1.66	**0.11**	0.64	0.57	21.49	1.13	**0.27**
Life stage (Adult)	-1.26	0.68	21.62	-1.85	**0.08**	-1.61	0.64	21.47	-2.52	**0.02**
**Random effects**	*σ^2*	*S.D.*				*σ^2*	*S.D.*			
UniqueTrial_no	1.40	1.18				1.30	1.14			
Residual	2.82	1.68				1.95	1.40			
Observations	233					230				
N(UniqueTrial_no)	25					25				
Marginal R^2 / Conditional R^2	0.14 / 0.43					0.18 / 0.51				
